# Repeat multiparametric MRI in prostate cancer patients on active surveillance

**DOI:** 10.1371/journal.pone.0189272

**Published:** 2017-12-27

**Authors:** Juho T. Eineluoto, Petrus Järvinen, Anu Kenttämies, Tuomas P. Kilpeläinen, Hanna Vasarainen, Kevin Sandeman, Andrew Erickson, Tuomas Mirtti, Antti Rannikko

**Affiliations:** 1 Department of Urology, University of Helsinki and Helsinki University Hospital, Helsinki, Finland; 2 Medical Imaging Center, University of Helsinki and Helsinki University Hospital, Helsinki, Finland; 3 Department of Pathology (HUSLAB), University of Helsinki and Helsinki University Hospital, Helsinki, Finland; 4 Finnish Institute for Molecular Medicine, University of Helsinki and Helsinki University Hospital, Helsinki, Finland; 5 Medicum, University of Helsinki and Helsinki University Hospital, Helsinki, Finland; University of Chicago, UNITED STATES

## Abstract

**Introduction:**

This study was conducted to describe the changes in repeat multiparametric MRI (mpMRI) occurring in prostate cancer (PCa) patients during active surveillance (AS), and to study possible associations between mpMRI-related parameters in predicting prostate biopsy (Bx) Gleason score (GS) upgrading >3+3 and protocol-based treatment change (TC).

**Materials and methods:**

The study cohort consisted of 76 AS patients with GS 3+3 PCa and at least two consecutive mpMRIs of the prostate performed between 2006–2015. Patients were followed according to the Prostate Cancer Research International Active Surveillance (PRIAS) protocol and an additional mpMRI. The primary end points were GS upgrading (GU) (>3+3) in protocol-based Bxs and protocol-based TC.

**Results:**

Out of 76 patients, 53 (69%) had progression (PIRADS upgrade, size increase or new lesion[s]), while 18 (24%) had radiologically stable disease, and 5 (7%) had regression (PIRADS or size decrease, disappearance of lesion[s]) in repeat mpMRIs during AS. PIRADS scores of 4–5 in the initial mpMRI were associated with GU (p = 0.008) and protocol-based TC (p = 0.009). Tumour progression on repeat mpMRIs was associated with TC (p = 0.045) but not with GU (p = 1.00). PIRADS scores of 4–5 predict GU (sensitivity 0.80 [95% confidence interval (CI); 0.51–0.95, specificity 0.62 [95% CI; 0.52–0.77]) with PPV and NPV values of 0.34 (95% CI; 0.21–0.55) and 0.93 (95% CI; 0.80–0.98), respectively.

**Conclusion:**

mpMRI is a useful tool not only to select but also to monitor PCa patients on AS.

## Introduction

Prostate cancer (PCa) is the most common male cancer [1]. The mortality of PCa in Europe is the third highest among all cancers in men, with 73 000 deaths in 2015 [2]. The majority of PCa patients, however, do not die of the disease but harbour clinically insignificant disease, which does not affect life expectancy even if left untreated. These patients are candidates for active surveillance (AS). The aim of AS is to reduce or postpone treatment-related side effects by monitoring the disease and offering timely, curative treatment when needed. Inclusion criteria for AS vary worldwide, but typically patients included in AS have the following: a small, focal cancer with low Gleason score (GS), only a few positive cores in systematic 12-core transrectal ultrasound (TRUS)-guided prostate biopsies and a low prostate-specific antigen (PSA) concentration [3]. Most surveillance protocols rely on repeated biopsies (Bx), PSA measurements and digital rectal examination to detect possible disease reclassification or true biological progression. Histological findings of Bx remain the main prognostic factor for PCa. However, random Bx may repeatedly miss significant cancers of the prostate, which contributes to undergrading of the PCa present in the patient [4]. AS cohort studies with radical prostatectomy (RP) or confirmatory Bx samples show an upgrading from diagnostic Bx for 20 to 30 percent of the cases [3,5].

Multiparametric MRI (mpMRI) and MRI-targeted fusion biopsies (FBx) have the potential to select patients for AS and to assist in clinical decision making when indicated [6]. However, little is known about the role of mpMRI as a follow-up tool during AS [7]. Therefore, we sought to characterise the changes occurring in repeated mpMRI in PCa patients on AS.

The specific purposes of this study were 1) to characterise changes occurring in repeat mpMRI during AS, and 2) to study possible associations between mpMRI-related parameters, and changes thereof, in predicting Bx GS upgrading >3+3 (GU) and protocol-based treatment change (TC).

## Materials and methods

**Fig 1:**
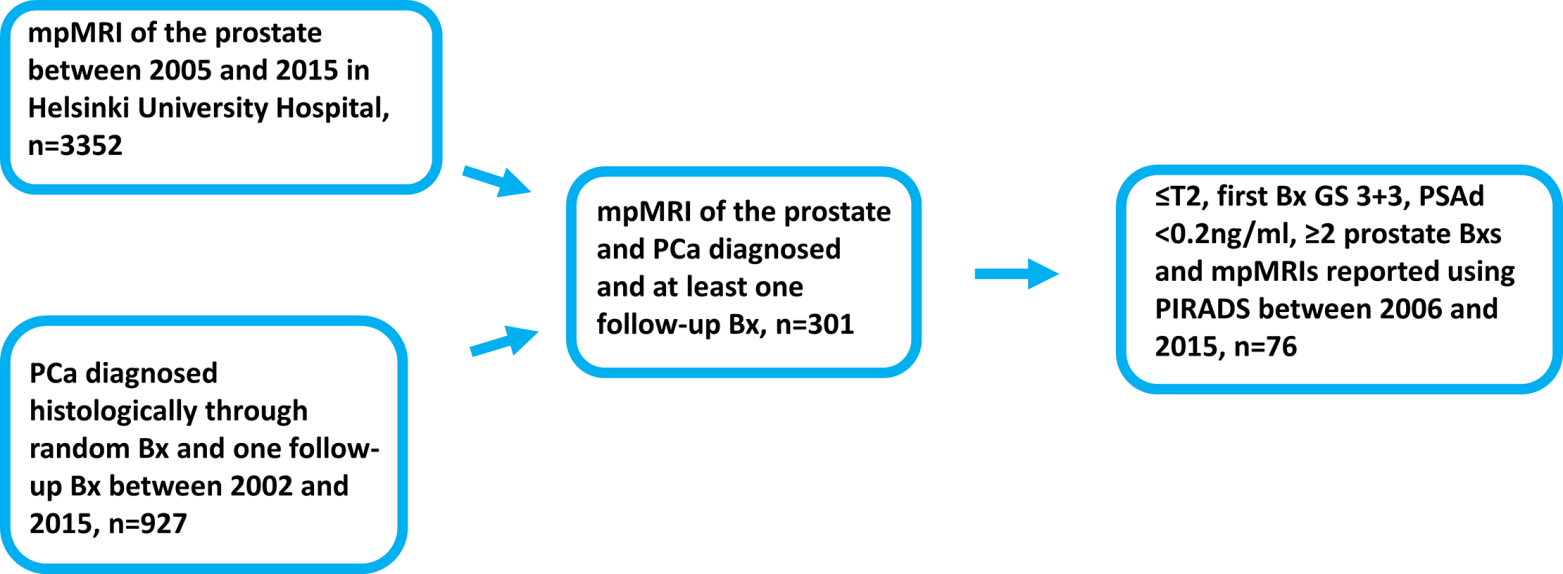
Selection of the 76 active surveillance patients. Abbreviations: mpMRI = Multiparametric Magnetic Resonance Imaging; Bx = Prostate biopsy; PCa = Prostate cancer; PIRADS = Prostate Imaging Reporting And Data System 1.0; GS = Gleason score. PSAd = Prostate specific antigen density.

The study approval by the Institutional Ethics Committee of the Helsinki University Hospital (diary number 386/13/03/02/2014) waived the need for consent based on the retrospective nature of the study and on the research conducted using only the local registry data and images. The study’s population selection is presented in Fig 1. Briefly, all PCa patients who underwent prostate MRI in the Helsinki University Hospital (HUCH) between January 2005 and May 2015 (n = 3352) and patients on AS with at least one repeat Bx between January 2002 and April 2015 (n = 927) were identified and linked. A chart review was performed for the 76 patients, who were identified as having GS 6 disease and having had at least two prostate MRIs performed according to the Prostate Imaging Reporting and Data System version 1 (PIRADS) [8]. The AS inclusion criteria were histologically proven diagnosis of PCa, clinical stage ≤ T2, GS ≤ 6 and PSA density ≤ 0.2 ng/ml. One patient had prostatitis at the time of his PCa diagnosis but was included in the analysis despite high PSA and PSA density due to a transient rise in these values. After diagnosis, patients on AS were followed according to the Prostate Cancer Research International Active Surveillance (PRIAS) protocol [9,10]. Cognitively targeted Bx were often taken along with random Bxs if PRIAS protocol-based triggers were reached, e.g. PSA-DT <10 years.

The mpMRI protocol can be found here http://dx.doi.org/10.17504/protocols.io.j66crhe. The majority of the patients had their first mpMRI within the first initial diagnostic year on AS, i.e. during the first year of diagnosis before the confirmatory Bx, which was scheduled according to the PRIAS protocol. Repeat mpMRIs were not systematically performed but repeated on most patients within the first two years on AS and thereafter whenever deemed clinically necessary (often when PSA DT <10 years). The mpMRI parameters included T2WI, DWI with ADC mapping and DCE. Imaging was performed between April 2007 and May 2015 using a 3.0 T Philips Achieva MRI scanner with 4 mm (all sequences until 2012) or 3 mm (T2WI from 2013 on) slices. The MRI protocol evolved over the study period according to then current knowledge. Following their publication in May 2012, the European Society of Urogenital Radiology (ESUR) guidelines for mpMRI were followed [11]. For instance, the highest b-value used in DWI for tumour detection was raised from 600 to 800 in June 2012, and finally to 2000 in September 2013. Moreover, signal intensity curves became available after the implementation of ESUR prostate MRI guidelines. However, contrast-enhanced images without dynamic curves were available throughout the entire period. Every parameter was scored according to PIRADS v1 criteria by four experienced uroradiologists, each having at least five years of experience in interpreting prostate MRIs. Inter-reader agreement was not evaluated. A total of 58 patients had at least one of their mpMRIs prior to the PIRADS era, and retrospective reading of these scans was done according to PIRADS v1 by one uroradiologist (AK) who was blinded to the subsequent clinical data of the patients. The overall PIRADS score was compiled using T2 as the primary determining sequence in the transition zone and DWI in the peripheral zone. Tumour size was measured in three dimensions (AP x CC x LAT x 0.5) on T2WI. Pathological progression was defined as a GS above 6 in any of the subsequent protocol-based Bxs during AS. TC was defined as ≥3 positive Bx, clinical stage ≥T3 or GS >3+3, i.e. any event that discontinued AS based on the PRIAS protocol. PSA DT was not included in the analysis as a covariate, as it is used as a surrogate trigger for disease reclassification in the PRIAS protocol. mpMRIs with PIRADS scores of 3, 4 and 5 were considered positive, whereas PIRADS scores below 3 were considered negative. Each lesion identified in mpMRI was separately scored according to PIRADS. When the patient had multiple suspicious lesions, the lesion with the highest PIRADS score was included in the analysis. In cases where there was more than one lesion with the same PIRADS score, the largest lesion was included in the analysis. The performance of mpMRI was analysed by determining the sensitivity, specificity, positive predictive value (PPV) and negative predictive value (NPV) of the first and the second mpMRI in relation to pathological progression. mpMRI progression was defined as an increase in PIRADS score of the previously existing lesions or the appearance of new lesion(s) or an increase of ≥0.1cm^3^ in tumour volume. mpMRI regression was defined as a decrease in PIRADS score or the disappearance of lesion(s) or a decrease of ≥0.1cm^3^ in tumour volume in subsequent mpMRIs. Pearson’s χ^2^ test of independence and Cramer’s V were performed to examine the association between categorical variables. All analyses were performed using SPSS® Statistics software (IBM® Version 21). A two-tailed p-value of less than 0.05 was considered statistically significant.

## Results

The study’s population demographics and mpMRI data are detailed in Tables 1 and 2. The median interval between the first and second mpMRIs was 24 months (range 4–73 months) for all patients, 14 months (range 4–72 months) for patients who underwent TC and 41 months (range 5–73 months) for those who continued AS. Altogether 171 MRIs were performed. Of the 76 patients, 50 (65.8%) had at least one positive mpMRI (PIRADS score 3–5). Furthermore, 35 out of 76 patients (46.1%) had a high PIRADS suspicion with scores of 4 or 5 on at least one occasion.

**Table 1:** Demographics of the study cohort.

	(range or % of total)
**Patients**	76 (100)
**Median age, years**	64 (41–77)
**Median PSA at diagnosis, ng/ml**	5.6 (1.1–17.4)
**Median free PSA at diagnosis, %**	12.0 (6.0–36.0)
**Median follow-up time, months**	66 (11–112)
**Median time between mpMRIs, months**	24 (4–73)
**Histologic Bx upgrading, number of patients**	15 (20)
** of which GS 3+4 = 7**	12 (16)
** of which GS 4+3 = 7**	3 (4)
**Median time between diagnostic and**	
** latest Bx (GS = 6), months, n = 61**	48 (11–86)
** upgrading Bx (GS >6), n = 15**	41 (12–85)

Abbreviations: PSA = Prostate Specific Antigen; Bx = Prostate Biopsy; GS = Gleason Score, mpMRI = Multiparametric Magnetic Resonance Imaging.

**Table 2:** Comparison of two mpMRIs of 76 prostate cancer active surveillance patients.

	First mpMRI n = 76	Second mpMRI n = 76
**Median prostate volume, cm^3^**	40.0 (15–80)	40.0 (20–120)
**Median number of lesions**	1 (0–2)	1 (0–3)
**Median size of lesions, cm^3^**	0.15 (0–5.2)	0.20 (0–3.7)
**No suspicious lesions on mpMRI, number of patients**	33 (43)	34 (45)
**PIRADS score 1**	0 (0)	0 (0)
**PIRADS score 2**	4 (5)	1 (1)
**PIRADS score 3**	24 (32)	14 (18)
**PIRADS score 4**	8 (11)	8 (11)
**PIRADS score 5**	7 (9)	19 (25)
**Positive mpMRI (PIRADS 3–5), number of patients**	38 (50)	41 (54)

(range or % of total); Abbreviations: mpMRI = Multiparametric Magnetic Resonance Imaging; PIRADS = Prostate Imaging Reporting And Data System 1.0.

**Fig 2:**
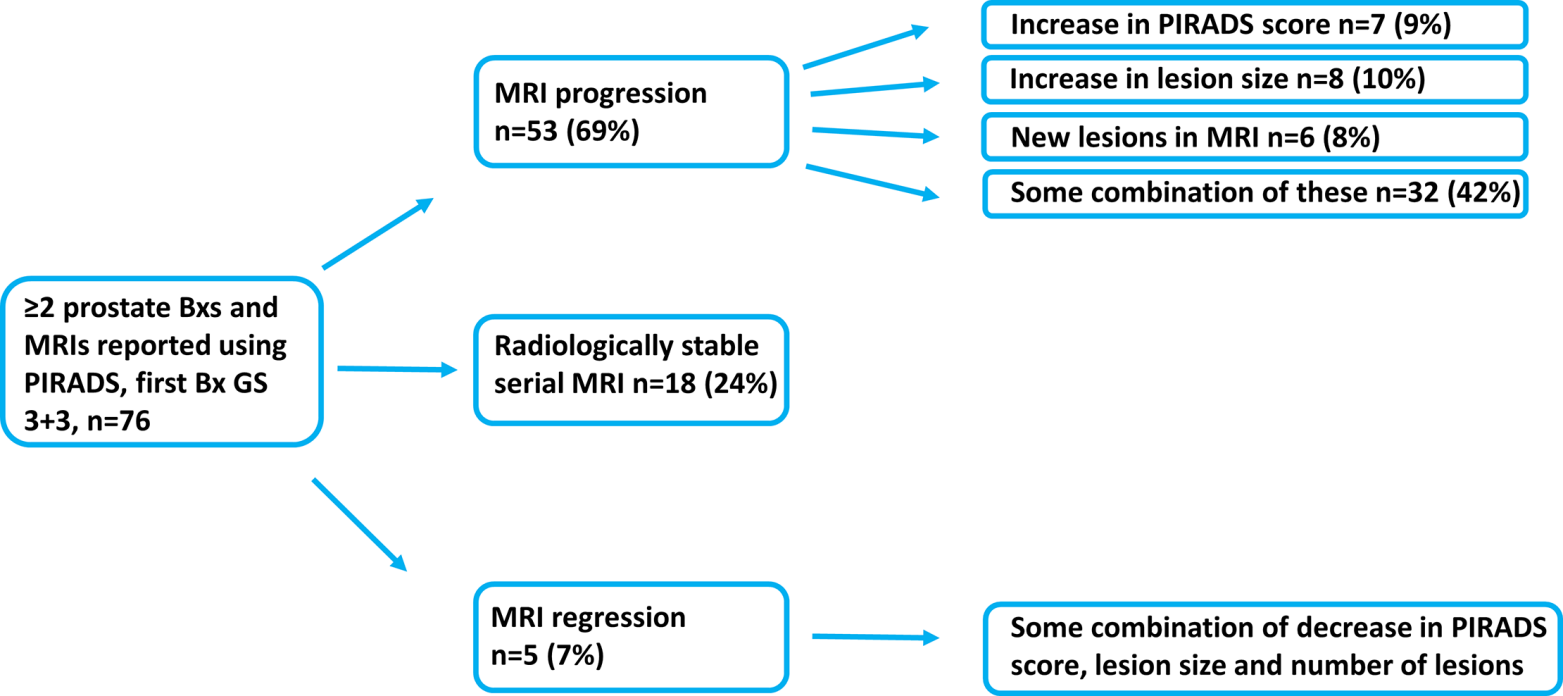
Serial mpMRI results of the 76 AS patients. Abbreviations: mpMRI = Multiparametric Magnetic Resonance Imaging; PIRADS = Prostate Imaging And Data Reporting System 1.0; Bx = Biopsy; AS = Active surveillance.

**Fig 3:**
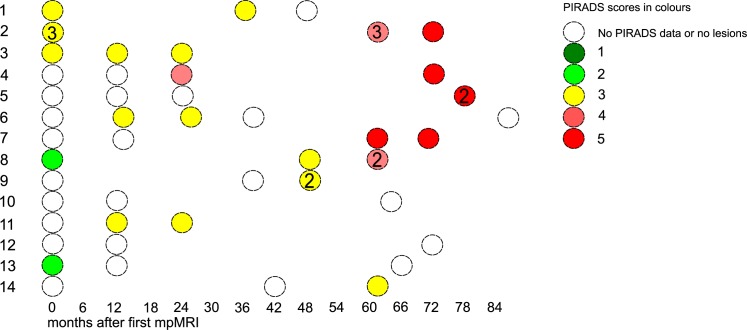
Characteristics of serial mpMRI changes in 14 patients with ≥3 mpMRIs. Horizontal axis shows time from first mpMRI. Each ball refers to a single mpMRI. Color refers to PIRADS score. Number refers to number of suspicious lesions.

Fig 2 depicts the mpMRI results of the 76 AS patients. Out of the 76 patients, 53 (69%) had mpMRI progression in subsequent mpMRIs: 7 (9%) had an increase in PIRADS score only, 8 (10%) had an increase in lesion size only, 6 (8%) had new lesions, and 32 (42%) had some combination of these. Radiologically stable disease was observed in 18 (24%) of the 76 patients. Only 5 (7%) of the 76 patients had mpMRI regression in subsequent mpMRIs (a combination of decrease in lesion number, size and PIRADS score). Further data on the mpMRIs are shown in Table 3. The association between mpMRI progression and GU was not statistically significant (χ^2^ = 0.12; φ = 0.039; p = 1.0) whereas the moderate association between mpMRI progression and TC was statistically significant (χ^2^ = 4.0; φ = 0.23; p = 0.045). PIRADS scores of 4–5 in the first mpMRI strongly associated with TC (χ^2^ = 6.8; φ = 0.30; p = 0.009) and GU (χ^2^ = 8.6; φ = 0.34; p = 0.008). PIRADS scores of 3–5 in the first mpMRI did not associate with TC (χ^2^ = 3.5; φ = 0.22; p = 0.069) or GU (χ^2^ = 1.8; φ = 0.15; p = 0.18). PIRADS scores of 4–5 predicted GU with a sensitivity of 0.80 (95% confidence interval (CI); 0.51–0.95), specificity of 0.62 (95% CI; 0.52–0.77), and PPV and NPV values of 0.34 (95% CI; 0.21–0.55) and 0.93 (95% CI; 0.80–0.98), respectively. GU ≥7 in protocol-based random Bxs occurred in 15 out of 76 patients (19.7%). Fig 3 illustrates the changes occurring during surveillance in 14 patients with ≥3 mpMRIs.

**Table 3:** Serial mpMRI change (rise in tumour size, number or volume) and protocol-based treatment change. Correlations.

	**mpMRI progression negative**	**mpMRI progression positive**	**Total**	**p-value**
**Treatment change negative**	17	26	43	0.045
**Treatment change positive**	6	27	33	
**Total**	23	53	76	
**PIRADS scores 4–5 in the first mpMRI and Gleason Score upgrading**				
	**No PIRADS scores 4–5**	**PIRADS scores 4–5**	**Total**	**p-value**
**GU negative**	53	8	61	0.008
**GU positive**	8	7	15	
**Total**	61	15	76	

Abbreviations: mpMRI = Multiparametric Magnetic Resonance Imaging; GU = Gleason Score Upgrading ≥3+3; PIRADS = Prostate Imaging And Data System 1.0.

## Discussion

Over two-thirds (69%) of the patients with initially very low-risk PCa showed progression on serial mpMRI during AS. Tumour progression on serial mpMRI was also associated with treatment change from AS to definitive treatment. Furthermore, AS patients with a negative mpMRI were very unlikely to show histological upgrading in follow-up Bx. These results suggest that mpMRI is a valuable tool for PCa risk stratification not only at diagnosis but also during AS. In addition, the PIRADS scoring system seems to be effective in assessing prostate mpMRI changes.

The major limitations of the study are its retrospective nature and lack of a standardised Bx protocol based on mpMRI findings. Cognitively targeted Bxs were taken in addition to the systematic Bxs within the sextants that contained tumour as according to the mpMRI. The FBx technique was not available when the first patients, included in this study, were diagnosed, and, therefore, additional targeted cores were occasionally taken. However, due to the retrospective nature of the study, location and accompanying data for these cores cannot be accurately assessed. In 2015, our Bx protocol changed and MRI-ultrasound-FBxs [12] from patients with PIRADS lesions of 3–5 began to be performed. Furthermore, during the study period there occurred significant technological developments in prostate MRI scanning methods. For example, there were substantial changes in the b-values used for DWI-MRI. Therefore, early mpMRIs with lower b-values are not fully representative by current standards. The later mpMRI protocols with higher b-values (i.e. better tumour detection) have probably affected mpMRI progression as assessed by the appearance of new lesions. On the other hand, tumour size has been measured in T2 sequences, and which method remained largely unchanged throughout the study period. Therefore, while changes in mpMRI protocol reflect the rapid evolution in the field of prostate MRI, we believe that these changes have not had a major impact on the interpretation of the results. We note that the reporting of prostate mpMRI is currently undergoing rapid transformation, as emphasised by a recent update of the new PIRADS scoring system from version 1 to version 2, and the fact that the preparation of version 3 is already on the horizon [13].

The strengths of the study include the use of the categorised, structured PIRADS scoring system for suspicious lesions in mpMRI, the use of a well-defined protocol for AS (PRIAS) and relatively long intervals between MRIs. Our early experience with the use of prostate MRI scans in an AS cohort prior to the PIRADS era was recently published and it suggested a poor correlation between MRI and GU [14]. The data presented in the current series, however, are in sharp contrast to our previous findings. To support repeatability, ESUR prostate mpMRI guidelines were published in 2012. The fact that our cohort spans a long period of time during which there occurred technological changes in MRI, including the emergence of the PIRADS reporting system, likely explains the observed differences. It has been shown that mpMRIs reported according to the PIRADS protocol show more accuracy in cancer detection, especially of the higher risk tumours [7,15]. Moreover, prostate mpMRI reports given in a structured format according to PIRADS classifications, rather than subjective terminology, is likely to be of greater value to a clinician [7,15]. This not only explains the differences between our previous report and the current data, but also highlights the importance of structured mpMRI evaluation and reporting.

**Fig 4:**
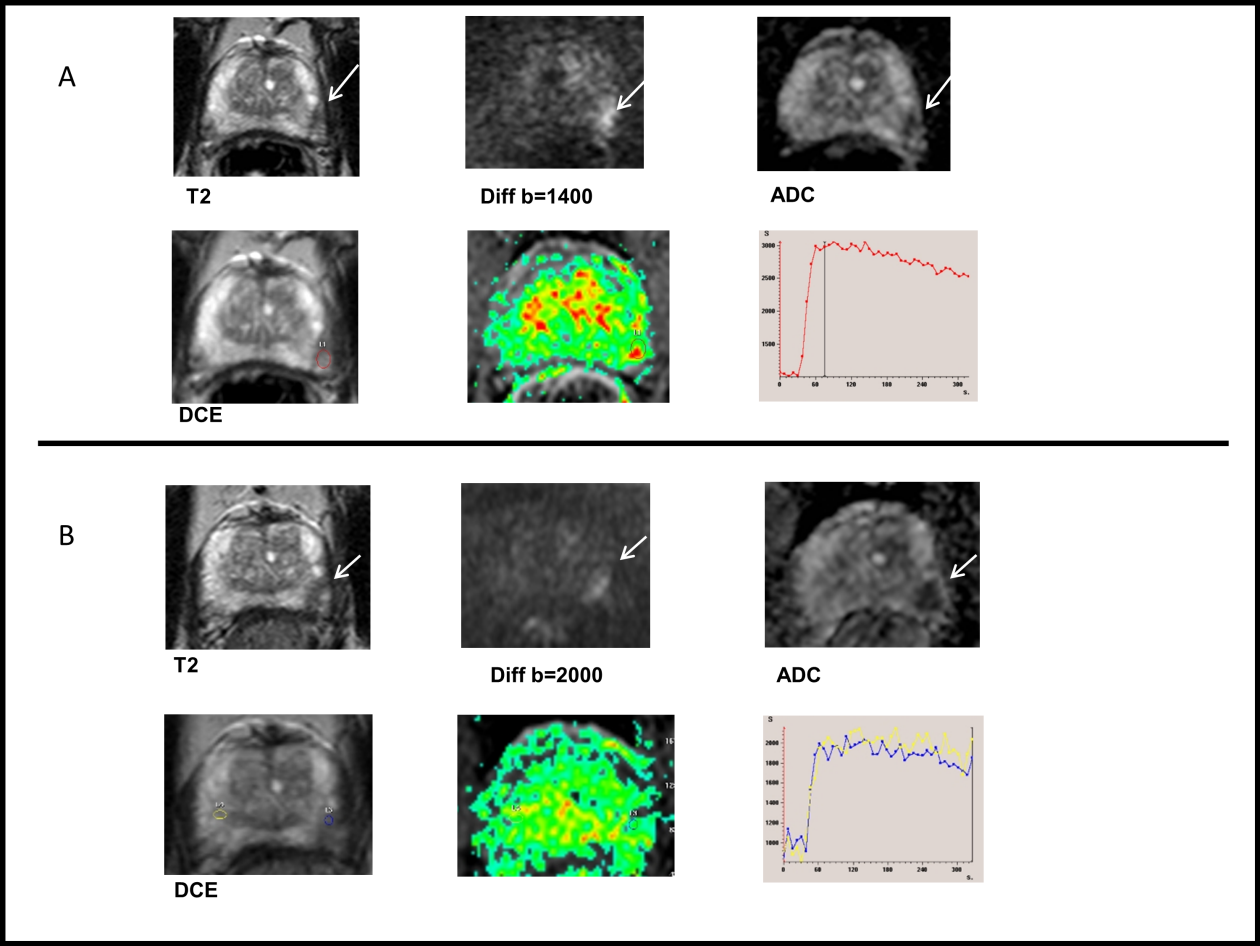
An example of tumour progression. **(A)** Prostate MRI taken in September 2013. 71-year-old patient in AS. T2WI shows a small low signal intensity lesion (max diameter 9 mm). Restricted diffusion and focal enhancement was found in left midgland. Routine biopsies revealed Gleason score (GS) 3+3 prostate cancer in left apex and right base. (**B)** Same patient, prostate MRI taken in January 2015. The lesion appeared larger (max diameter 13 mm) in T2WI and more visible in diffusion and ADC map, while DCE remained unspecific. GS 4+5 prostate cancer was found in cognitively targeted biopsies. Radical prostatectomy in June 2015 proved GS 4+3 disease with two small foci in the right lobe as well. Also, 0.5 mm extraprostatic extension with 5 mm contact to external tissues was diagnosed. Tumour stage was pT3a.

Our PCa patient population, with two mpMRIs, was highly preselected towards a very low-risk patient cohort. Patients with large and high-grade tumours are more likely to be detected earlier during surveillance, and to subsequently receive active treatment. Accordingly, patients available for the second mpMRI have a low risk for large GS >6 disease. However, 69% of the 76 patients showed progression on mpMRI during surveillance, even though the remaining AS cohort likely represents a cohort of men with only “negligible” risk PCa that remains after implementing PRIAS follow-up protocol. This suggests that current PRIAS protocol, mainly relying on PSA-DT and random Bx, is not sufficient to identify patients at risk of harbouring clinically significant disease. A similar discontinuation rate was recently observed in a large AS Movember Global Action Plan (GAP3) cohort consisting of more than 14 000 patients. This highlights the importance of proper follow-up during AS even if very low-risk patients are selected for AS. It also reflects our inability to predict PCa behaviour accurately at diagnosis using traditional clinical tools. The selection bias may also explain the lack of association between mpMRI change and GU, as more sophisticated targeting methods may be needed to detect the remaining smaller GS >6 cancer foci. Fig 4A and 4B depict tumour growth among patients with ≥3 mpMRIs. The criteria for both progression and regression were arbitrary and subjective as no standardised criteria exists. Therefore, we focused upon the significance of observed changes in mpMRI in lieu of setting exact cut-off values for judging tumour growth. Furthermore, possible infections and inflammation, often encountered in patients in AS cohorts that rely on frequent repeat Bxs, need to be taken into consideration when interpreting prostate MRI in the AS setting.

We have previously shown that adherence to repeat Bxs in the PRIAS cohort is greatly attenuated by Bx-related complications such as infections [16,17]. The current PRIAS protocol recommends an mpMRI instead of TC for patients with PSA-DT <3 years, as PSA-DT is a sensitive but insufficiently specific marker for PCa progression. Accordingly, prostate mpMRI seems to be an attractive substitute for Bxs, especially due to its high NPV. This finding concurs with the findings of other studies on non-AS cohorts [7,18] and AS cohorts [19–21]. When NPV is high, there is little chance of a clinically significant PCa occurring if the mpMRI shows no suspicious lesion [7,18,22]. However, the follow-up times of all cohorts, published up to the present time, are relatively short. A longer follow-up may reveal clinically significant PCa missed by mpMRI. Furthermore, the methods used as the gold standard in calculating the NPV are standard Bx, targeted Bx or RP specimen findings. These are all inadequate in terms of reliable comparator (Bxs fail to detect clinically significant disease and RP lacks true negatives).

All the currently published cohorts demonstrate that treatment-free survival curves are far from optimum, which is mainly due to diagnostic inaccuracy, i.e. routine diagnostics fail to detect a substantial proportion of GS >3+3 cancers as do the follow-up protocols. In this respect, our data further support the use of mpMRI not only at diagnosis (in accurately identifying patients that have the possibility of clinically significant disease), but also as a tool for monitoring patients during AS. Therefore, an appropriate aim would be to more accurately select patients for early change in their plan of treatment and perhaps to simplify and personalise subsequent follow-up. Data on serial mpMRI among AS patients are scarce and suffer from a lack of standardisation for definition; this is particularly problematic in progressing prostate mpMRI. Recently, Diaz et al. and Felker et al. presented similar cohorts of PCa patients with serial mpMRI data [19,20]. While their data are comparable to ours, all three studies suffer from the aforementioned lack of standardisation.

Our finding that low PIRADS scores were less useful for detecting high GS PCa is consistent with findings reported by others [7,15]. This may eventually alter the use of Bx in AS, as the use of mpMRI becomes more common and the standard practice. Recent studies found that mpMRI-assisted targeted FBxs show greater accuracy in the detection of clinically significant PCa, with a complimentary reduction in the proportion of patients diagnosed with clinically insignificant PCas [18,20,22]. Thus, the use of mpMRI and MRI-guided applications are likely to become more commonplace. At the same time, factors other than histological grading alone (e.g. biomarkers) could be expected to become more important in the avoiding overtreatment of slowly progressing disease. The results of our study support the role of mpMRI as a tool to aid in deciding on whether to continue AS or undertake active treatment. Our results show that mpMRI missed only 1 out of 15 (6.7%) cancers with GS ≥7, which yielded a high NPV. However, the use of mpMRI should be prospectively incorporated into AS trials to study the potential benefit in a randomised setting, as in the current Nordic SPCG-17 trial (http://clinicaltrials.gov, NCT02914873).

## Conclusion

Prostate mpMRI is a useful tool not only for guiding patient selection for AS at diagnosis, but also for monitoring during surveillance.

## Supporting information

S1 File Dataset. The data behind the statistics.
(TXT)
